# Evaluation of High-Throughput PCR and Microarray-Based Assay in Conjunction with Automated DNA Extraction Instruments for Diagnosis of Sepsis

**DOI:** 10.1371/journal.pone.0026655

**Published:** 2011-11-22

**Authors:** Sanna Laakso, Juha Kirveskari, Päivi Tissari, Minna Mäki

**Affiliations:** 1 Mobidiag Ltd, Helsinki, Finland; 2 Helsinki University Hospital Laboratory (HUSLAB), Department of Bacteriology, Helsinki, Finland; Charité-University Medicine Berlin, Germany

## Abstract

**Background:**

High incidence of septic patients increases the pressure of faster and more reliable bacterial identification methods to adapt patient management towards focused and effective treatment options. The aim of this study was to assess two automated DNA extraction solutions with the PCR and microarray-based assay to enable rapid and reliable detection and speciation of causative agents in the diagnosis of sepsis.

**Methodology/Principal Findings:**

We evaluated two automated DNA instruments NucliSENS® easyMAG® and NorDiag Arrow for the preparation of blood culture samples. A set of 91 samples flagged as positive during incubation was analyzed prospectively with the high-throughput generation of Prove-it™ Sepsis assay designed to identify over 60 Gram-negative and Gram-positive bacterial species as well as methicillin resistance marker from a blood culture. Bacterial findings were accurately reported from 77 blood culture samples, whereas 14 samples were reported as negative, containing bacteria not belonging to the pathogen panel of the assay. No difference was observed between the performance of NorDiag Arrow or NucliSENS® easyMAG® with regard to the result reporting of Prove-it™ Sepsis. In addition, we also assessed the quality and quantity of DNA extracted from the clinical *Escherichia coli* isolate with DNA extraction instruments. We observed only minor differences between the two instruments.

**Conclusions:**

Use of automated and standardized sample preparation methods together with rapid, multiplex pathogen detection offers a strategy to speed up reliably the diagnostics of septic patients. Both tested DNA extraction devices were shown to be feasible for blood culture samples and the Prove-it™ Sepsis assay, providing an accurate identification of pathogen within 4,5 hours when the detected pathogen was in the repertoire of the test.

## Introduction

Sepsis is a life-threatening disease, associated with high rates of morbidity and mortality. It is estimated that 4 million out of 13 million septic patients die worldwide each year [1]. Time to diagnosis for sepsis and an early initiation of effective antimicrobial therapy has been a major predictor of an outcome of a septic patient [2], [3], [4]. Kumar and colleagues (2006) demonstrated a strong relationship between the delay in initiation of antimicrobial therapy and reduced survival. The risk for death in septic patients increased 7,6% per hour after the first six hours of documented hypotension. Thus, an early identification of the causative agent is crucial and often informative enough for directing treatment decisions towards an evidence-based antimicrobial therapy [5], [6].

Currently, blood culture is the gold standard of diagnosis for sepsis. The method is based on cultivation and detection of viable micro-organisms present in blood. The presumptive pathogen classification from a positive blood culture is first concluded on the basis of morphological features and cell wall characteristics of the microbe by Gram-staining. Positive blood culture is subcultured further on different growth media and a set of phenotypic tests are run for a characterization and identification of the microbe. The definitive identification of pathogens is usually achieved within one to three days after the blood culture is flagged as positive, but may take even longer for atypical and fastidious organisms. Culture is at the moment the only possibility for determining the antimicrobial susceptibility of the pathogen. These methods are time-consuming and highly manual procedures, which delay efficient, pathogen-driven patient management [7], [8], [9].

Many novel molecular strategies have emerged to speed up diagnosis for sepsis. One of the recently introduced approaches is a combination of PCR and microarray. The main advantages of microarray over other DNA-based approaches are broad pathogen coverage, the potential to differentiate closely related microbial species accurately, and simultaneous identification of multiple microbes in the single reaction [10], [11], [12].

The Prove-it™ Sepsis assay, consisting of a broad-range PCR and microarray-based platform (Mobidiag, Finland) has been recently evaluated in the clinical setting using over 3300 positive blood cultures. Tissari and co-workers (2010) [13] indicated the assay to be 99% specific and 95% sensitive, with a pathogen panel covering over 50 clinically relevant bacterial species as well as the methicillin resistance marker. They also concluded that the assay was on average one day faster than the culture-based gold standard and could thus enable earlier evidence-based management for clinical sepsis. The newest generation of the assay is the high-throughput Prove-it™ StripArray platform having 8 well strips and containing one microarray at the bottom of each well. This microarray platform allows parallel analysis of 1 to 96 samples in one run.

When adapting a PCR-based protocol in a routine clinical setting, a prerequisite for a sensitive analysis is the efficient preanalytical sample preparation step, including DNA extraction. Hence, these steps should always be evaluated carefully, especially in respect to cell wall disruption of a microbe and subsequent recovery of microbial DNA without putative PCR inhibitors originated from the clinical sample. In recent years, a number of DNA extraction instruments have become available to response to the needs of faster and labor-efficient solutions. Automated DNA extraction allows for simultaneous preparation of a high number of samples with reduced hands-on time and human errors, thus improving precision, reproducibility and traceability [14], [15], [16], [17].

The aim of the study was to bring together novel technologies for faster sepsis diagnostics; automated DNA extraction to be used in conjunction with the PCR and microarray-based bacterial identification method. We evaluated the Prove-it™ Sepsis StripArray platform together with *in vitro* diagnostic labeled NucliSENS®easyMAG® (bioMérieux, France) and NorDiag Arrow (NorDiag, Norway) extraction instruments. These platforms utilize magnetic particle based extraction technology. Both instruments were also compared for their relative efficiency in recovering and purifying bacterial DNA from the clinical *E.coli* isolate and their technical aspects of usability.

## Results

### Evaluation of DNA extraction instruments using blood culture samples

We evaluated the functionality and suitability of the NucliSENS®easyMAG® and NorDiag Arrow extraction instruments for sepsis diagnostics together with the Prove-it™ Sepsis assay, using positive blood culture material. In total, 91 samples flagged as positive during the blood culture incubation from patients suspected with sepsis were collected in two weeks time in October, 2009. The samples were not consecutive. Tissari and co-workers (2010) extracted DNA from over 3300 blood culture samples, using the NucliSENS®easyMAG® instruments with the starting volume of 100 µl and the elution volume of 55 µl. These volumes were also used in this study, but the volumes of NorDiag Arrow were adjusted according to the recommendations of the used Arrow VIRAL NA kit, using the starting volume of 250 µl and the elution volume of 100 µl.

The DNA extracts of blood culture samples were analyzed with the Prove-it™ Sepsis assay. The PCR and microarray-based results showed the perfect concordance in bacterial identifications between DNA extracts of both instruments (Table 1). Among the 91 positive blood culture samples, 41 (45,1%) Gram-negative and 32 (35,2%) Gram-positive bacteria were detected. In addition, four (4,4%) polybacterial and 14 (15,4%) negative samples were reported. The most commonly identified bacteria were *Escherichia coli* (29,7%), coagulase negative staphylococci other than *Staphylococcus epidermidis* (CNS, 9,9%), *S. epidermidis* (6,6%), and *Klebsiella pneumoniae* (5,5%). The negative results were achieved from non-target pathogens, such as *Streptococcus viridans* and *anginosus* group, *Moraxella osloensis*, *Capnocytophaga canimorsus*, and *Micrococcus* sp. The bacteria found represent well the overall distribution of blood culture findings in this laboratory.

**Table 1:** Comparison of identifications of various agents identified from the blood culture (BC) samples with respect to the used DNA extraction solutions of NucliSENS®easyMAG® and NorDiag Arrow.

Identified bacteria by Prove-it™Advisor	Number of samples	Propotion of BC samples (%)	Concordance of bacteria identifications between DNA extracts of NucliSENS®easyMAG® and NorDiag Arrow (%)
**Gram-negative bacteria**			
*Bacteroides fragilis* group	2	2,2	100
*Enterobacter cloacae*	1	1,1	100
*Enterobacteriaceae* group	2	2,2	100
*Escherichia coli*	27	29,7	100
*Haemophilus influenzae*	1	1,1	100
*Klebsiella peumoniae*	5	5,5	100
*Proteus mirabilis*	1	1,1	100
*Pseudomonas aeruginosa*	1	1,1	100
*Serratia marcescens*	1	1,1	100
**TOTAL**	**41**	**45,1**	
**Gram-positive bacteria**			
CNS	9	9,9	100
*Enterococcus faecalis*	3	3,3	100
*Propionibacterium acnes*	1	1,1	100
*Staphylococcus aureus*	4	4,4	100
*Staphylococcus epidermidis*	6	6,6	100
*Streptococcus agalactiae*	2	2,2	100
*Streptococcus dysgalactiae* subsp. *equisimilis*	3	3,3	100
*Streptococcus pneumoniae*	4	4,4	100
**TOTAL**	**32**	**35,2**	
**Polybacterial identifications**			
*Enterococcus faecalis, Enterococcus gallinarum, Proteus vulgaris*	1	1,1	100
*Enterococcus faecalis, Staphylococcus epidermidis*	1	1,1	100
*Staphylococcus aureus,* CNS	1	1,1	100
*Staphylococcus aureus, Staphylococcus epidermidis*	1	1,1	100
**TOTAL**	**4**	**4,4**	
**No organism detected**			
Negative	14	15,4	100
**TOTAL**	**91**	**100**	

### Correlation between Prove-it™ Sepsis and the reference results

We compared the bacterial identification results of blood culture samples analyzed with the Prove-it™ Sepsis assay to those of the reference method. Overall, the results between the two methods were similar. In case of 12 conflicting results (Table 2), the blood culture samples were subjected further to test repeating, DNA sequencing and/or more data were obtained from HUSLAB in order to study more closely the bacterial species in question.

**Table 2:** Identification discrepancies of blood culture samples between the reference method and Prove-it™ Sepsis categorized on the basis of reported results.

Reported results			
Prove-it™Sepsis	Reference method	Number of samples	Confirmed speciation
*Enterobacteriaceae* group	*Escherichia coli*	2	*Enterobacteriaceae* group
*Enterococcus faecalis, Enterococcus gallinarum, Proteus vulgaris*	*Enterococcus faecalis, Enterococcus gallinarum/casseliflavus, Proteus vulgaris, Enterococcus faecium*	1	*Enterococcus faecalis, Enterococcus gallinarum, Proteus vulgaris, Enterococcus faecium*
*Enterococcus faecalis, Staphylococcus epidermidis*	*Staphylococcus epidermidis*	1	*Staphylococcus epidermidis,*
Negative	CNS, *Streptococcus viridans* group	1	*Staphylococcus capitis, Streptococcus viridans* group
*Staphylococcus aureus,* CNS	CNS	1	*Staphylococcus capitis*
*Staphylococcus epidermidis*	CNS	2	*Staphylococcus epidermidis*
*Streptococcus dysgalactiae* subsp. *equisimilis* *	*Streptococcus pyogenes*	2	*Streptococcus dysgalactiae* subsp. *equisimilis* *
*Streptococcus pneumoniae*	Negative	2	*Streptococcus pneumoniae*
**TOTAL**		**12**	

*Streptococcus dysgalactiae subsp. *equisimilis* expressing also the Lancefield's serogroup A.

In one pair of aerobic and anaerobic bottles from the reference method *E. coli* was reported, whereas the PCR and microarray assay reported *Enterobacteriaceae* group. We sequenced the 5′region of the *gyrB* gene of these samples and conducted homology searches and sequence comparisons against the public (EBI and NCBI) and proprietary sequence databases. The sequences showed relative 96% (381/398 bp) similarity to the *gyrB* sequence of *E.coli*, but the similarity was not enough to make a definitive conclusion of the species found. Two samples from the same patient that were reported as *Streptococcus pyogenes* by the reference method shared 100% homology with the *gyrB* gene region of *Streptococcus dysgalactiae* subsp. *equisimilis* which was reported by the PCR and microarray assay. The strain, however, represented Lancefield group A antigen, which is by definition the prerequisite for indentifying *S. pyogenes*. The sequencing also confirmed the PCR and microarray-based speciation of two *Staphylococcus epidermidis* samples which were originally identified as CNS. From one sample, the reference method identified CNS when the PCR and microarray assay reported a negative result. The sequencing result specified the CNS species to be *Staphylococcus capitis,* which was not included in the CNS taxon of the PCR and microarray assay; therefore, the reported results were regarded as concordant. *Streptococcus pneumoniae* was identified from two samples, two anaerobic bottles, by the PCR and microarray assay, when the reference method reported negative result in the other sample and *S. pneumoniae* was detected by Accuprobe hybridization test (GenProbe, USA) from the other sample. The confirmatory probe hybridisation test was performed due to pathognomonic staining result; poorly stained, autolysed coccoid bacterial structures detected both in Gram-staining and acridine orange staining.

In three samples, the PCR and microarray-based results were not completely accurate. From one polybacterial sample, the assay identified organisms *Enterococcus faecalis, Enterococcus gallinarum, Proteus vulgaris*, but failed to detect *Enterococcus faecium*. *Staphylococcus aureus* and CNS were reported from one sample, whilst the reference method reported only CNS. The sequencing specified CNS species to be *Staphylococcus capitis.* Most probably, the strain variation and excess amount of amplicons led to cross-hybridization with *S.aureus* oligonucleotide probes, resulting in false positive identification of *S. aureus.* Contamination due to human error also caused one false positive identification of *Enterococcus faecalis* by the PCR and microarray assay.

On the basis of bacterial identification and additional data comparison, including sequencing results, we concluded that 96.7% (88/91) of the samples were speciated correctly by the Prove-it™ Sepsis assay, including 14 negatively reported findings from non-target pathogens.

### Analysis of DNA yield and purity

Three subsamples of *E. coli* suspensions with the assigned OD_600_ values of 3, 2, 1, 0.1, and 0.01 were extracted parallel with NucliSENS®easyMAG® and NorDiag Arrow, after which the yields and purity (A_260/280_) of DNA extracts were compared by the means of spectrophotometer and real-time PCR analysis.

**Figure 1:**
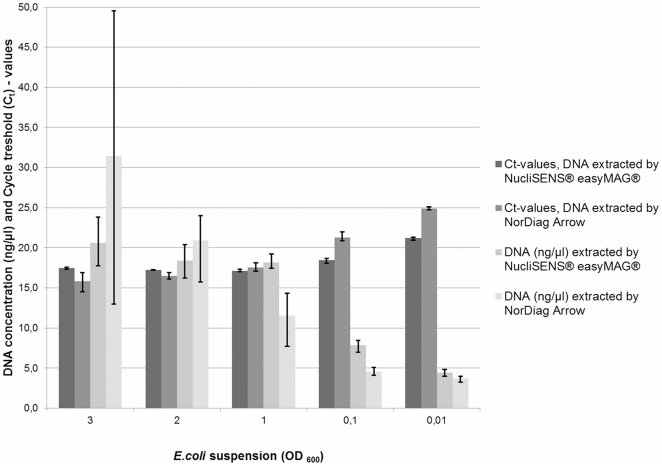
Comparison of the DNA yields of extracted *E.coli* DNA. Average values and their standard deviations of the three replicated subsamples of *E.coli* DNA extracts are presented in the columns. Columns are classified by the optical density values (OD_600_) of the *E.coli* suspensions measured prior to DNA extraction. DNA concentrations (ng/µl) were measured by a spectrophotometer and Cycle treshold (C_t_) -values are based on the real-time PCR.

DNA yields from the samples with a higher cell density, *i.e.* samples with the assigned OD_600_ values of 3, 2, and 1 were measured to be between 15–30 ng/µl by a spectrophometer, whereas the samples with a lower cell density, *i.e.* samples with the assigned OD_600_ values of 0.1 and 0.01 were measured to be ∼5 ng/µl. The means of DNA concentrations from the three parallel subsamples extracted with the both instruments were approximately at the same level (Figure 1). However, we found differences between the tested DNA extraction instruments when we compared the standard deviations between triplicates from the assigned OD_600_ values of 3, 2, 1, 0.1, and 0.01. DNA concentrations from the triplicates varied slightly when DNA extraction was performed with NorDiag Arrow.

**Figure 2:**
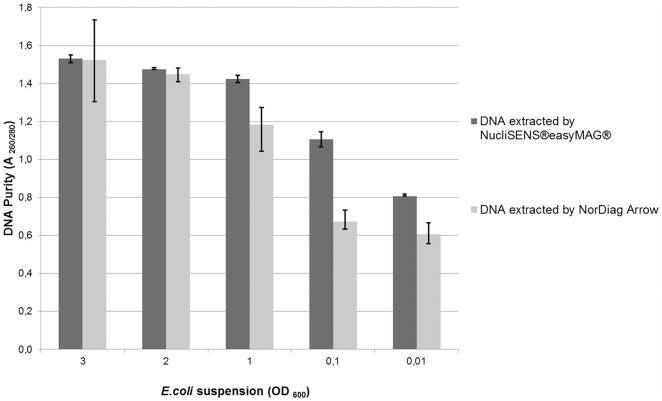
Comparison of the purity (A _260/280_) of extracted *E.coli* DNA. Average values and their standard deviations of the three replicated subsamples of *E.coli* DNA extracts are presented in the columns. Columns are classified by the optical density values (OD_600_) of the *E.coli* suspensions measured prior to DNA extraction. Purity of the extracts was determined by a spectrophotometer.

On average, the A_260/280_ ratios of 1,2 to1,5 were measured for the samples with the assigned OD_600_ values of 3, 2, and 1 and the A_260/280_ ratios of 0,6 to 1,1 for the samples with the assigned OD_600_ values of 0.1 and 0.01 (Figure 2). The similar variation in standard deviations as detected with DNA yields was also found when we examined the purity of the triplicate samples extracted with NorDiag Arrow. In contrast, triplicates extracted with the NucliSENS®easyMAG® showed smaller deviations in the measured values of DNA concentration and purity.

The DNA extracts were also subjected to the real-time PCR of the *E. coli* chromosomal d-1-deoxyxylulose 5-phosphate synthase gene (*dxs*) [18] for the comparison of the cycle threshold (C_t_)-values. In general, higher C_t_-values reaching over 20 were associated with lower DNA concentrations (∼5 ng/µl) measured by a spectrophotometer and *vice versa* with higher DNA concentrations of 15–30 ng/µl associated with C_t_-values below 20. DNA concentrations measured by a spectrophotometer showed a wider range than the C_t_-values obtained during real-time PCR runs. Thus, the similar kind of deviations obtained by a spectrophotometer was not found from the C_t_-values. Otherwise, the yield measurements and C_t_-values of real-time PCR were in accordance.

### Technical comparison of the NucliSENS®easyMAG® and NorDiag Arrow instruments

The NucliSENS®easyMAG® and NorDiag Arrow instruments were also compared for the technical aspects. The technical analysis included the comparison of flexibility in sample and elution volumes, a total DNA extraction time and the number of sample per run (Table 3). The analysis was conducted based on the kits and the programs used in this study.

**Table 3:** Comparative analysis of the technical aspects of NucliSENS®easyMAG® and NorDiag Arrow.

	aNucliSENS®easyMAG® (bioMérieux)	bNorDiag Arrow (NorDiag)
**Samples per one run**	1–24	1–12
**Total turnaround time (min)** (including lysis step, excluding hands-on time)	50 (10 min for lysis step, 40 min for extraction step)	58
**Sample volume (µl)**	10 to 1000	250, 550
**Eluation volume (µl)**	25 to 110, in 5 µl increments	100

a Generic 2.0.1 protocol.

b Arrow VIRAL NA kit and the Viral 010 program.

## Discussion

In this study, we assessed the performance of a newest generation of Prove-it™ Sepsis assay, incorporating the bacterial detection of proven performance [13] into the high-throughput platform. The accurate identification was demonstrated by analyzing 91 positive blood culture samples. We compared the obtained PCR and microarray-based results with those of the reference method, observing only 12 (13,2%) conflicting identifications, of which DNA sequencing confirmed nine PCR and microarray results. Three remaining discrepant results were either due to contamination while processing the sample, or inaccurate result reporting due to possible cross-hybridization or missing reporting of *E. faecium* from the polybacterial samples, consisting of *E. faecalis, E. faecium, E. gallinarum,* and *P. vulgaris*. For other polybacterial samples, the identifications were correct. When the additional information and sequencing results were taken into account we concluded that the bacterial speciations with the PCR and microarray were correct in 96,7% of the analyzed samples when the analysis was limited to those bacteria covered by the assay. Similar figure has been detected in earlier studies [13], [19].

Of note was that *S. pyogenes* was identified from two samples of one patient by the reference method, but the PCR and microarray assay reported *S. dysgalactiae* subsp. *equisimilis* findings from both samples, which were also confirmed by the sequencing. Pyogenic streptococci are divided into serogroups based on their Lancefield group antigens. *S. dysgalactiae* subsp. *equisimilis* usually exhibits the Lancefield's serogroup C or G antigens, whereas *S. pyogenes* exhibits the serogroup A. The used reference method exploited the serotyping of streptococci in the species identification. Similar results to those in this study were also noticed by Brandt and co-workers (1999) [20], who studied in detail the clinical blood culture isolates of *S. dysgalactiae* subsp. *equisimilis* expressing also the Lancefield's serogroup A. These isolates share virulence determinants with *S.pyogenes*, thus emphasizing the importance of both Lancefield grouping as well as biochemical data. The DNA-based methods may be of assistance in accurate speciation of beta-hemolytic streptococci.

DNA-based methods are also preferred in cases when blood culture samples are reported as false negatives due to the autolysis of microbes or in the case of fastidious organisms. This was most likely the case when *S. pneumoniae* was identified by the PCR and microarray assay, but a negative result was reported by the reference method. *S. pneumoniae* is known to have a tendency to undergo autolysis when it reaches the stationary phase of growth [21] and therefore, it cannot always be reported by the conventional blood culture-based method.

We studied the isolates identified as *E.coli* by the reference method and *Enterobacteriaceae* by the PCR and microarray assay in great detail. The sequencing of the conserved 5′ gene region of topoisomerase gene *gyrB* showed 96% homology to that of *E. coli*, showing sequence variation at many nucleotide positions untypical for *E.coli*. We also sequenced the *gyrB* gene regions of *Escherichia fergusonii* and *Escherichia hermannii,* possessing similar phenotypic characters as *E.coli,* and also related to misidentification of *E.coli* [22]. However, the sequences of these species were not similar with the isolate in question. Also, the homology searches against the sequence databases did not reveal higher homology to any other bacterial species. Therefore, we concluded that the isolate could belong to the *Escherichia* taxon, but was not *E. coli.* In the study of Tissari and colleague (2010), also four similar blood culture isolates with 97–98% homology with the *E. coli gyrB* sequence were observed (data not published). Although these results are interesting in terms of bacterial taxonomy, we acknowledge that in these cases the misidentification of *E. coli* would have little or no clinical significance.

In response to recognized limitations of the current gold standard method of sepsis diagnostics, several new diagnostic strategies have been emerged [23]. The requirements for new clinical diagnostics of sepsis include automated, labor-efficient solutions that provide accurate diagnostic results in a timely manner. Typically, DNA-based technologies involve three consecutive steps: nucleic acid extraction, amplification and detection together with data analysis. Various automated nucleic acid extraction devices have been spread to the field of DNA-based sepsis diagnostics [24]. The performance of the DNA extraction solution impacts on the sensitivity and success of subsequent downstream analysis. In this study, we brought together two aspects of novel technologies for sepsis diagnostics; automated sample preparation together with multiplex DNA-based identification of causative bacterial agents.

We evaluated the performance of two automated DNA extraction instruments NucliSENS®easyMAG® and NorDiag Arrow for the preparation of blood culture samples prior to the Prove-it™ Sepsis analysis. Both DNA extraction instruments efficiently lysed the microbes and seemed to remove the possible PCR inhibitors. The PCR and microarray-based results obtained from the DNA extracts of blood culture samples were in perfect accordance when the results from the two instruments were compared. The bacteria from which DNA were extracted consisted of nine clinically relevant Gram-negative and 10 Gram-positive bacterial species. Among the Gram-positive bacteria were commonly encountered species in sepsis, such as *Staphylococcus* and *Streptococcus* spp, possessed a rigid cell wall which can be difficult to lyse [25].

In addition to the qualitative bacterial identification comparison, we also conducted quantitative analysis by comparing yields and purity of DNA extracts of clinical isolate of *E.coli*. DNA yields of the eluates of both instruments were comparable in samples originated from the same *E. coli* suspension. However, minor deviations were found in the concentrations of the eluates of three parallel samples extracted with NorDiag Arrow. The variability of the standard deviations was wider when the concentrations were measured by a spectrophotometer, but such a variation was not found so strongly from C_t_-values determined by a real-time PCR assay. In general, the A_260/280_ ratios of the measured DNA extracts did not reach the optimal purity value of 1.8. We noticed that the low cell density in the starting volume prior to the extraction correlated in lower A_260/280_ ratios values measured. A_260/280_-ratios are linearly reduced by the increasing protein concentration and other contaminants putatively found in DNA extracts, having absorbance in the region 230 nm to 320 nm. The measurement of the DNA concentration out in the optimal purity range of DNA, *i.e.* the A_260/280_-ratios of 1.5–2.0, should be regarded only as suggestive [26]. Furthermore, we cannot rule out the limitations of the spectrophotometer measurements.

The other perspective of usability of the extraction instruments to the diagnostic procedures is to take a short view for the NucliSENS®easyMAG® and NorDiag Arrows technical aspects (Table 3). In general, advantages of the NucliSENS®easyMAG® are flexibility, like highly adjustable starting and elution volumes and capability to handling various sample types with the same reagent kit. Also, 1–24 samples can be run simultaneously in the instrument. However, depending on the number of samples analyzed in a routine basis, a smaller bench-top instrument capable of running up to 12 samples, like NorDiag Arrow can be sufficient enough for diagnostics, especially if the instrument provides cost-efficient purchasing and running costs. The turn-around times of the instrument were similar, less than one hour. We considered that the platforms were robust, easy-to-use and require little hands-on-time, allowing more effective use of resources.

In conclusion, NucliSENS®easyMAG® and NorDiag Arrow DNA extraction devices were shown to be efficient and feasible for preparation of blood culture samples when used in conjunction with the Prove-it™ Sepsis assay. The accurate identification of pathogens was available within 4,5 hours in the same day as the blood culture flagged as positive. New diagnostics assays are needed for the prompt and reliable identification of causative agents of sepsis in order to avoid delays in administration of appropriate, pathogen-driven antimicrobial therapy. This study shows that an integration of automated and standardized sample preparation method together with a PCR and microarray-based assay into patient management pathways provides the solution that decreases hands-on-time while increasing accuracy, robustness, and traceability. Moreover, the combination offers faster diagnosis of septic patients than the current conventional culture-based diagnostics, which in turn has the potential to lead to positive outcomes.

## Materials and Methods

### Samples

#### Blood culture material and reference method

A total of 91 blood culture samples flagged as positive from patients with suspected sepsis were collected in the Department of Bacteriology, HUSLAB, Finland, during the two weeks of October in 2009. Blood samples taken into aerobic and anaerobic blood culture bottles of BacT/Alert® (bioMérieux, France) were incubated in the blood culturing instrument BacT/ALERT 3 D (bioMérieux) for up to 5–6 days until they were reported as negative if no sign of microbial growth was detected. If possible growth was observed, the microbe was conventionally speciated and the results represent the reference method in this study. Gram-staining, and when necessary, acridine orange staining, were performed on all flagged samples. Biochemical tests for bacterial identification and antibiotic susceptibility testing were performed according to the CLSI guidelines (http://www.clsi.org). These analyses were primarily performed directly from an aliquot of the blood culture sample. Additional biochemical or molecular biology tests were performed when necessary. Typically, it took one to two days to identify the microbe from a positive blood culture.

#### Clinical isolate

Clinical isolate of Escherichia coli, provided by HUSLAB, was aerobically cultured on a blood agar for 24 h at 37°C. Cultured bacteria were suspended to the 1× phosphate buffered saline (PBS) buffer (Jena Bioscience GmbH, Germany) and the optical density (OD) of a bacterial suspension at 600 nm was measured by a spectrophotometer (BioPhotometer, Eppendorf). Bacterial suspensions with assigned OD_600_ values of 3, 2, 1, 0.1 and 0.01 were prepared. Each suspension was divided into three parallel subsamples (triplicates) prior to DNA extraction.

### DNA extraction

DNA extraction was performed using both NucliSENS®easyMAG® (www.biomerieux.com) and NorDiag Arrow (www.nordiag.com) instruments. NucliSENS®easyMAG® was used according to the Generic 2.0.1 protocol, having a starting volume of 100 µl and an elution volume of 55 µl with the blood culture material. In parallel, NorDiag Arrow was used according to the Arrow VIRAL NA kit and the Viral 010 program, having a starting volume of 250 µl and an elution volume of 100 µl. In case of clinical isolates, the starting volume was 250 µl and the elution volume was 100 µl with the both instruments.

### PCR and microarray assay

DNA extracts of blood culture samples were analyzed by the Prove-it™ Sepsis StripArray assay (Mobidiag, Finland), according to the manufacturer's instructions. Briefly, the Prove-it™ Sepsis assay was based on broad-range PCR and microarray technologies and designed to identify bacterial pathogens in positive blood cultures [13], [19]. The proprietary primers were used for the amplification of specific regions of the bacterial topoisomerase genes *gyrB* and *parE* [27], and the methicillin resistance gene *mecA*. For the amplification step, the Mastercycler® epgradient S thermal cycler (Eppendorf, Germany) was used.

The amplicons were subsequently introduced onto the microarray area of the Prove-it™ StripArray. Positive hybridization-based reactions were detected and reported by the StripArray Reader and the Prove-it™ Advisor analysis software (version 1.0). The target identification was interpreted using specific built-in rules and parameters of the Prove-it™ Advisor software. Prior to the result reporting, the internal control probes of the assay, evaluating mainly the functionality of the PCR and hybridization steps were required to pass the built-in rules. The result consisted of the name(s) of identified bacterial target(s) and detailed information about data, such as signal intensities and number of identified oligonucleotide probes. The DNA extraction and PCR controls included in each test series were required to be negative for the acceptance of a particular test series.

### DNA sequencing

For sequencing, various sets of specific primers originated from *gyrB* gene region were applied. Sequencing was performed using cycle sequencing with Big Dye Terminator kit (version 3.1, Applied Biosystems, USA) and reactions were run on ABI 3130xl capillary sequencer according to the manufacturer's instructions. For sequence homology searches, BLAST algorithm [28] against the European Bioinformatics database (www.ebi.ac.uk/Tools), the National Center for Biotechnology Information database (blast.ncbi.nlm.nih.gov/Blast.cgi), and the proprietary sequence database of Mobidiag was applied. The definitions of the bacterial species were made on the bases of the sequence homology with the known bacterial species of these used databases.

### Quality and quantity determination

After parallel DNA extraction with NucliSENS®easyMAG® and NorDiag Arrow, quality of subsamples of *E.coli* suspensions (OD_600_ values of 3, 2, 1, 0.1, and 0.01) were studied by spectrophotometer measurements and real-time PCR. 1 µl of extracted DNA was pipetted to the NanoDrop spectrophotometer (Thermo Fisher Scientific, USA), which reported the DNA concentration and purity of nucleic acids (A_260/280_). The real-time PCR designed for the *E. coli* chromosomal d-1-deoxyxylulose 5-phosphate synthase gene (*dxs*) [18] was conducted, applying a SYBR Green I dye –based assay and Stratagene Mx3000P Q-PCR System with Stratagene MxPro Q-PCR Software, version 4.10 (Agilent Technologies, USA). The PCR mixture was prepared using Brilliant II SYBR Green Q-PCR kit (Agilent Technologies, USA): 1× Brilliant II SYBR Green Q-PCR master mix, 30 nM Reference dye, forward primer F (5′-CGAGAAACTGGCGATCCTTA-3′) and reverse primer R (5′-CTTCATCAAGCGGTTTCACA-3′) at a final concentration of 1 µM (Metabion, Germany), 2 µl of isolated DNA and PCR-grade water to bring total volume to 15 µl. The thermal cycling protocol was as follows: initial denaturation for 10 min at 95°C followed by 40 cycles of 15 sec at 95°C, 1 min at 54°C and 1 min at 72°C, continuing to the one cycle of 15 sec at 95°C, 1 min at 60°C and 15 sec at 95°C for the dissociation curve analysis. At the end of the run, the software produced the dissociation curve and amplification plots together with the cycle threshold (C_t_) -values for the further analysis.
